# Cost-Benefit Analysis of the Upland-Rice Root Architecture in Relation to Phosphate: 3D Simulations Highlight the Importance of S-Type Lateral Roots for Reducing the Pay-Off Time

**DOI:** 10.3389/fpls.2021.641835

**Published:** 2021-03-12

**Authors:** Daniel Gonzalez, Johannes Postma, Matthias Wissuwa

**Affiliations:** ^1^Graduate School of Agriculture and Life Sciences, The University of Tokyo, Tokyo, Japan; ^2^Crop, Livestock, and Environment Division, Japan International Research Center for Agricultural Sciences, Tsukuba, Japan; ^3^Forschungszentrum Jülich GmbH, Institute of Bio- and Geoscience – IBG-2: Plant Science, Jülich, Germany

**Keywords:** OpenSimRoot, *Oryza sativa* (L.), phosphate uptake, L-type lateral roots, root branching, root hairs, modeling

## Abstract

The rice root system develops a large number of nodal roots from which two types of lateral roots branch out, large L-types and fine S-types, the latter being unique to the species. All roots including S-types are covered by root hairs. To what extent these fine structures contribute to phosphate (P) uptake under P deficiency was investigated using a novel 3-D root growth model that treats root hairs as individual structures with their own Michaelis-Menten uptake kinetics. Model simulations indicated that nodal roots contribute most to P uptake followed by L-type lateral roots and S-type laterals and root hairs. This is due to the much larger root surface area of thicker nodal roots. This thickness, however, also meant that the investment in terms of P needed for producing nodal roots was very large. Simulations relating P costs and time needed to recover that cost through P uptake suggest that producing nodal roots represents a considerable burden to a P-starved plant, with more than 20 times longer pay-off time compared to S-type laterals and root hairs. We estimated that the P cost of these fine root structures is low enough to be recovered within a day of their formation. These results expose a dilemma in terms of optimizing root system architecture to overcome P deficiency: P uptake could be maximized by developing more nodal root tissue, but when P is growth-limiting, adding more nodal root tissue represents an inefficient use of the limiting factor P. In order to improve adaption to P deficiency in rice breeding two complementary strategies seem to exist: (1) decreasing the cost or pay-off time of nodal roots and (2) increase the biomass allocation to S-type roots and root hairs. To what extent genotypic variation exists within the rice gene pool for either strategy should be investigated.

## Introduction

Rice consumption in Africa is increasing rapidly, leading to a widening gap between demand and local rice production (Nigatu et al., 2017). One reason for this widening gap is the relatively low rice productivity in Sub-Saharan Africa (SSA), which is due to a combination of low production inputs and unfavorable production environments (Saito et al., 2019). About 30% of the rice cropping area in Africa is in the more stress-prone uplands (Diagne et al., 2013) where irrigation is nor provided. The average upland rice yields in Africa are around 1.2 t ha^–1^ (Drame and Manneh, 2013) compared to an average of 4.75 t ha^–1^ in irrigated rice in Asia in 2016 (IRRI World Rice Statistics, 2020).

African farmers producing upland rice are generally low-income farmers that acquire very limited amounts of fertilizers and other inputs (Tsujimoto et al., 2019). Their economic conditions exacerbate problems inherent to the uplands, where highly weathered soils such as Oxisols tend to bind phosphate (P) in plant-unavailable forms. High P fixation in soils coupled with low P fertilization rates cause P to be the primary limiting factor in the production of rice in SSA (Saito et al., 2019). It is therefore of utmost importance that rice varieties with improved P acquisition and utilization efficiency are developed, as these provide a cost-efficient partial solution to the soil fertility problem in SSA (Vandamme et al., 2015).

Screening experiments have shown that genotypic differences exist for tolerance to P deficiency in the rice germplasm (Fageria et al., 2013; Vandamme et al., 2015) and that this can be caused by differences in P acquisition efficiency (Mori et al., 2016), P utilization efficiency (Wissuwa et al., 2015), or by a combination of both (Rose et al., 2013). P acquisition was found to mainly depend on differences in root system size with smaller effects contributed by differences in P uptake efficiency characterized by superior P uptake per unit root size (Mori et al., 2016; Wissuwa et al., 2020). One of the P deficiency tolerant rice genotypes combining vigorous seedling root growth with high P uptake efficiency is the gene bank accession ‘DJ123’ (Wissuwa et al., 2020). It originates from Bangladesh and belongs to the *aus* sub-species of rice that is well known as a source of donors for abiotic stress tolerance.

The size of a root system is the sum of its different components and in rice four main classes of roots are distinguished; main root axes are formed by the primary root and subsequent nodal roots (also called crown roots) and both classes give rise to two classes of lateral roots (Rebouillat et al., 2009). The larger L-type lateral roots contain several layers of cortex cells and typically have secondary or even tertiary branches while the small S-type lateral roots contain only one layer of cortical cells, are unbranched and short with a maximum of around 1 cm and a diameter smaller than 50 μm (Wissuwa et al., 2020). S-type laterals develop on both main axes and L-type laterals. They have so far only been reported for rice but not for other cereals. An order of magnitude finer than these S-type laterals are root hairs and recently Nestler et al. (2016) showed that these hairs develop on all root classes in rice, including on S-type roots, where they are present in high density but at slightly reduced length compared to hairs on the main axes. Experiments have shown that root hairs are directly involved in P uptake from the soil (Gahoonia and Nielsen, 1998) and that they are particularly effective in overcoming the combined effects of water and P deficiency stress (Brown et al., 2012). Considering that S-type lateral roots appear to be unique to rice, that these roots can contribute more than half of the total root system length (Nestler et al., 2016), and that they are covered by even finer root hairs, it becomes evident that rice has a particularly high proportion and amount of very fine root structures exploring the soil for resources like water and nutrients, which should be especially beneficial for poorly mobile nutrients like P.

What is not known is to what extent each root class and the hairs developing on them contribute to total plant P uptake. It would be of interest from a plant breeding point to estimate to what extent changes in properties of different root classes and hairs would improve P uptake, as this would allow for the formulation of clear breeding targets with relation to which specific root system trait would provide the largest improvements in P uptake. For example, using a modeling approach Zygalakis et al. (2011) predicted that increasing root hair length would improve P uptake more than increasing root hair density. Genotypic differences in root hair length have been reported for rice grown in soil and gene bank accession DJ123 was found to have slightly longer hairs compared to other genotypes (Nestler and Wissuwa, 2016). To what extent genotypes differ in S-type lateral root development has not been investigated in detail.

While mathematical models and computational simulations of rice and rice growth have been ongoing for almost 30 years (Morita and Abe, 1994), to our knowledge, modeling studies have not yet addressed the effect of differences in S-type length and density in the presence of root hairs.

Recently, De Bauw et al. (2020) presented a functional-structural model that computes water and P uptake from an upland rice variety, Nerica4, utilizing CRootBox. They concluded that S-type laterals would indeed contribute prominently to P uptake. Furthermore, their main conclusion was that the total number of root tips growing into previously undepleted soil are the main contributors to overall P uptake from a low-P soil and that L-type laterals and the secondary branching from these L-type laterals may contribute significantly to total root tip number. The model developed by De Bauw et al. (2020) successfully predicted water and P uptake in a P-fertilized soil, but underestimated P uptake from an unfertilized low-P soil. They hypothesized that this underestimation may be due to four factors; not accounting for the effect of mycorrhization, possible solubilization of immobile P by root exudates, overestimation of the Michaelis constant (Km), and additional P uptake contributed by root hairs.

Many of the current plant models have been developed and designed based on the results from experiments conducted in containers with relatively limited width. This has caused the issue of the root architecture being skewed toward a narrow and deep spread of roots. Analyses of rice root growth in the field has shown the opposite: a large proportion of roots are concentrated in the topsoil (first 15 cm), even for upland rice genotypes (Wissuwa et al., 2020). This was not only due to extensive root branching in the topsoil layer but also to considerable extension of crown roots horizontally (Mori et al., 2016). It is therefore imperative that model parameterization be developed based on field data, and not just greenhouse data. We consider that the difference in root spread and depth seen from different experimental setups can be significant, as P concentrations in the topsoil are higher compared to deeper soil layers (Mori et al., 2016).

With this in mind, we believe that an assessment of the influence of S-types, root hairs, and general positioning of roots on P uptake is necessary.

The lack of an upland rice model including all fine structures and a root distribution pattern not affected by spatial limitations in root proliferation enticed us to develop such a model. We decided to base our model on the P efficient breeding line DJ123 for several reasons, namely that its root system had been characterized in several prior studies which showed DJ123 to be similar to other rice genotypes for many root traits (Mori et al., 2016; Nestler and Wissuwa, 2016; Wissuwa et al., 2020); and that its efficient P uptake appears due to a combination of factors such as good early root vigor and high P uptake efficiency, rather than being caused by the extreme expression of a single root phenotype (Wissuwa et al., 2020). Specifically, the objectives of our study were (1) to obtain detailed data on root growth and P uptake of upland rice from both field and greenhouse experiments using DJ123 as a representative P-efficient upland rice genotype; (2) to use this data to parameterize and test a root architecture model that includes root hairs; (3) to use this root architecture model to simulate P uptake; (4) to conduct a sensitivity analysis in order to identify factors exerting a strong influence on P uptake; and (5) to conduct a cost-benefit analysis with regard to P needed to grow a certain root class *versus* the P uptake provided by that class.

## Materials and Methods

### Root Phenotyping Experiments

Parameters to describe root architecture and development were obtained for the upland rice genotype DJ123 from field and greenhouse experiments conducted in Tsukuba, Japan, and described in detail in Wissuwa et al. (2020). The soil was a highly P deficient (5 mg P kg^–1^ soil, Bray-II) volcanic ash soil (Humic Haplic Andosol). Plants were sampled from the field at 8, 20, 28, 48, and 62 days after emergence (DAE) to count crown roots per plant, and at 28 and 48 DAE entire root systems were excavated up to a depth of 30 cm. During this process, individual roots were traced and their coordinates in terms of length from crown to root tip and depth of the root tip were recorded and used to calculate root growth angles. The absence of container size limitations in the field allowed us to trace roots horizontally for up to 50 cm from their origin at the crown. Excavated roots were stored in water in zipped freezer bags in a refrigerator at 4°C until root scans were obtained. Shoots were dried and weighed as were roots after scans had been obtained. Tissue P analysis was done by digesting tissue samples in a 4:1 HNO_3_/HClO_4_ (nitric/perchloric) mixture followed by the determination of digest P concentrations in a microplate reader at 882 nm wavelength using the molybdenum blue assay defined by Murphy and Riley (1962).

A greenhouse experiment used the same field soil in containers of dimensions 23 × 46 × 15 cm depth (22-L) for harvests at 7 and 14 DAE or dimensions of 40 × 60 × 30 cm (72 L) for harvests at 21 and 28 DAE. Plants were sampled in weekly intervals by cutting off one side from the container and using a gentle water spray to remove the soil. This allowed us to excavate and scan intact root systems with minimum breakage. Afterward, the plants were treated and stored in the same manner as the excavated plants from the field experiments mentioned above.

### Root Scanning and Analysis

Roots were spread out in a 20 × 25 cm Perspex tray filled with 0.5 cm of and scanned using an Epson Perfection V700 photo dual-lens scanner with top lighting, with the following settings: 600 dpi, 8-bit grayscale, positive film. Obtained images were analyzed by the WinRhizo software (Version 2008a, Regent Instruments Inc) using a manual pixel classification with a gray-scale value of 225, to improve fine root detection. The debris filter was set to avoid counting debris with an area smaller than 1 cm^2^, and a length/width ratio smaller than 5. The root system was divided into diameter classes of: 0–90, 90–250, 250–400, 400–600, 600–1,000 nm, which correspond to S-type laterals (<90 nm), L-type laterals (90–250 nm) and crown roots (250–1,000 nm). Details on branching frequencies or length of individual lateral roots were obtained using ImageJ as described by Wissuwa et al. (2020). Micrographs obtained with an Olympus DP21 microscope (Olympus, Tokyo, Japan) with an Olympus UPlanApo lens at 4×, 10×, and 20× magnification were used to obtain exact measurements of root diameters.

### Modeling Root Growth in OpenSimRoot

We chose the publicly available software OpenSimRoot git version 6f8e57d63f (Postma et al., 2017) to develop a 3D root growth model of genotype DJ123. The modular nature of OpenSimRoot allows the creation of mini-models (objects that encapsulate a state variable) that are combined into modules that form a major model component (e.g., root branching or phosphorus uptake). This allows to easily add, remove, activate or deactivate components without having to rework the model in its entirety.

OpenSimRoot represents the root system by vertices and edges, with each root tip having its own vertex with dynamic coordinates, and as the root extends, vertices with fixed coordinates are placed behind it. Data required for this simulation are the location and time when root tips are created, the root growth rate, and the general direction of growth. In the initial step the primary root and hypocotyl emerge from the seed. Lateral roots emerge from parent roots depending on branching frequencies defined in space as an inter-branching distance (IBD) (Freschet et al., 2020). IBD is associated with the parent root class as the root tip of the parent root forms the primordia, whereas growth rates of laterals are defined by the lateral root class. Below, we summarize the main static and variable input parameters defining the root system development. For an extended explanation of the model, readers can refer to Postma et al. (2017).

• Rate of root primordia formation (day^–1^): This variable is defined for crown roots as the rate at which their creation from the hypocotyl occurs, and is dependent on the time that has passed since the seed was planted.
• Inter-branching distances (IBD, cm) define the distance between one branching lateral root and the next. To account for varying lateral root densities, we modeled IBDs based on parent-root-specific distributions, using normal distributions (means and standard deviations derived from experimental data) with truncated tails defined by maximum and/or minimum values. The model computes the rate of primordia formation from the IBD, using the parent root elongation rate at the time the primordia was formed. Thus, faster growing roots form more primordia per time. IBD input parameters are given in Supplementary Table 2. More frequent branching on the branched nodal roots was achieved manually through (i) modifying the IBD distribution of fast nodal roots to roughly double the IBD during the initial 2 weeks of root development, and (ii) to reduce subsequent branching frequencies since we observed that lateral root densities were higher in the basal half of the branched nodal roots.
• Growth rates (cm day^–1^): These are defined depending on the time since a root was created or branched (in days). Each root class has its own growth rate derived from observations from weekly samplings in the greenhouse experiment. The growth rate is slower initially (root emergence), reaches a maximum 2–10 days after creation, and slows down thereafter. Each root received a multiplication factor pooled from a (log) normal distribution such that roots of the same class vary in growth rate and final length (see Supplementary Table 3).
• General direction or growth: The initial growth direction is set by specified radial and axial branching angles. As the roots grow, the direction was changed by a tropism vector. The tropism vector is the combination of several vectors representing gravitropism, random impedance, and nutrient tropisms.
• Root diameter: The root diameters were set static for all root classes (0.065, 0.05, 0.06, 0.02, and 0.0045 cm for primary and fast nodal roots, branched nodal roots, nodal roots of tillers, L-type and, S-type lateral roots, respectively).
• Specific root volume (cm^3^ g^–1^): It was assumed to be static, as senescence and cell apoptosis was not considered in this model. Values were derived from measured root biomass and corresponding root volume data of WinRhizo for the entire root system. Values used were 0.08 g cm^–3^ for primary and nodal roots and this increased to 0.09 and 0.1 g cm^–3^ for L-type and S-type lateral roots, respectively. This increase is reflecting the decreasing proportion of aerenchyma in fine roots and was supported by the observation of increasing specific root volume in root systems with a lower proportion of nodal roots.
• Tillering: Rice tillering was also implemented in the model and tillers will produce nodal roots originating from the lowest tiller nodes.

In modeling root growth, we made defined parameters for the following root classes:

• Primary root (more highly branched compared to nodal roots).
• Nodal root branched.
• Nodal root fast (rapidly elongating nodal roots with less branching, increasing in frequency during later stages of development).
• Nodal roots of tillers (with a phenotype in between the other two nodal root classes).
• L-type lateral roots, with a distinction between branching off the primary root or nodal roots.
• S-type lateral roots, with a distinction between branching off the primary root *versus* branching off nodal roots and L-type lateral roots.

Root hairs develop on each of the above root classes but with varying root hair length and density between (i) primary root, (ii) nodal and L-type lateral roots, and (iii) S-type lateral roots (Nestler et al., 2016).

Making use of the modular nature of OpenSimRoot, we defined modules for growth of primary/nodal roots and L-type laterals, S-type laterals, and root hairs, and modules defining branching of L-type laterals, S-type laterals, and root hairs.

Root growth of genotype DJ123 was simulated over a 35-day period with a maximum timestep of 0.2 days for conditions of the highly P-fixing and therefore P deficient Andosol. Since P is considered the growth-limiting factor and since the root to shoot ratio is known to significantly increase in these conditions (Wissuwa and Ae, 2001; Fageria et al., 2013), we did not model root growth in dependence of photosynthetic carbon resources but on the observed growth of P-deficient DJ123 (Wissuwa et al., 2020). The soil was assumed to have a homogeneous distribution of P.

### Modeling P Uptake in OpenSimRoot

A radial advection-diffusion-reaction model was used to simulate P uptake rates (μmol cm^–1^ day^–1^) at individual root vertices. P-uptake rates were integrated over the length of the root system and time to get the total P uptake by the plant (Silberbush and Barber, 1983, 1984). P uptake is modeled for each individual root considering its length, radius, and presence of root hairs (at specific length and density). The soil parameters related to P availability were obtained based on analyses of the Andosol used in pot and field experiments and are summarized in Supplementary Table 1 (Rakotoson, unpublished data). Simulations based on these values significantly underestimated measured plant P uptake, despite the inclusion of root hairs and S-type laterals (see introduction). We presume that rice roots can solubilize small amounts of P in the rhizosphere to achieve this but did not simulate the solubilization *per se* as data describing the relevant processes is not available. To simulate the correct P uptake, we instead changed the initial P concentration in the soil solution from the measured 83 nM to 540 nM, which is still very low. For the buffer power (b) we used a value of 6,000 and the corresponding soil diffusion (De) was 9.227e^–6^ cm^2^ day^–1^ (Supplementary Table 1).

### Sensitivity Analysis and Model Simulations

A sensitivity analysis with five repeat runs was conducted with model base values altered by 25, 50, 100, 150, 200, and 400%. The analysis was conducted for two soil parameters (soil P concentration and buffer power) and six plant parameters: length and inter-branching distance for L-type and S-type roots, and length and density of root hairs. Effects of parameter variations on P uptake for a 28-day period were simulated.

We explored the cost-benefit aspects of different root classes. The weight of roots was calculated by the model as a function of root volume and specific densities of 0.06 g cm^–3^ for nodal roots, 0.07 g cm^–3^ for L-type roots, and 0.1 g cm^–3^ for S-type roots and root hairs. Different specific volumes (cm^3^/g) were assigned to root classes depending on the proportion of aerenchyma present in the respective class. The estimation of costs and benefits (P taken up) were simulated until day 31 (equal to 28 DAE in greenhouse/field experiments). Costs were estimated by multiplying simulated root class weight times the average root P concentration of DJ123 at 28 DAE (0.8 mg g^–1^). The cost recovery in days was estimated for three cases (see Table 3). In addition, simulations of cost-benefits were run for hypothetical root systems with shorter (0.5×) or longer (2.0×) S-type or L-type lateral roots and for reduced fast nodal root thickness (from 0.65 to 0.50 cm diameter).

## Results

### DJ123 Rice Root Architecture

**FIGURE 1:**
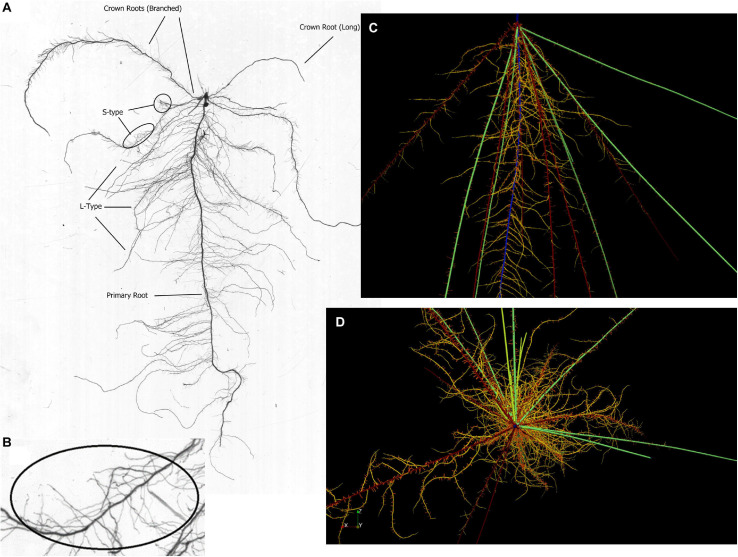
**(A)** Scan of a root system excavated 14 DAE from the greenhouse experiment showing the highly branched primary root as well as two nodal root classes and L-type and S-type lateral roots. Fast nodal roots start to develop between 7 and 14 DAE and therefore remain very small. **(B)** magnified region with a high density of S-type lateral roots. **(C)** Simulated root at 21 DAE where roots with their surrounding P depletion zones are shown for better visibility of lateral roots. Color coding identifies root classes: blue, primary root; dark red, branched nodal root; green, fast nodal root; yellow, L-type laterals; red, S-type laterals; light green, nodal roots from tillers. **(D)** Top-down view of a simulated root system at 28 DAE.qq.

Observations made during root excavations and in root scans led us to define 4 major root classes (primary root, crown roots, and L-type and S-type laterals), of which nodal roots were divided into three subclasses we termed branched nodal roots, fast nodal roots (faster elongating and less branched compared to the branched nodal roots), and nodal roots forming on tillers (in-between fast and branched nodal roots). The primary root was observed to be the most densely branched root with the longest lateral roots (Figure 1). The first nodal roots developing were of the branched class, being of intermediate length (35 cm at 28 DAE) with a growth rate of up to 1.5 cm day^–1^ and a root diameter between 340 and 650 μm (Table 1). Fast nodal roots started to develop between 7 and 14 DAE and increased in frequency as the root system matured. The length of the longest root excavated from the field at 28 DAE was 45 cm (Table 1). Assuming that the longest root was also the oldest one, we estimated it to be 21 days old and derived an average growth rate of 2.14 cm day^–1^. The diameter of the fast nodal roots was larger compared to branched nodal roots, ranging from 550 to 850 μm.

**TABLE 1:** Observed maximum root length, derived maximum daily growth rates, and typical ranges of root diameters observed on primary, nodal, and lateral roots.

Root type	Maximum length	Estimated^*x*^ growth rate	Diameter	Root hair density	Root hair length

	cm	cm day^–1^	μm	cm^–1^	cm
Primary root	46	2.19	500–700	1,400	0.02
Nodal root (branched)	35	1.46	340–650	1,400	0.015
Nodal root (long)	45	2.14	550–850	1,400	0.015
L-Type root	14.6	0.73	130–200	700	0.015
S-Type root	1.08	0.14	35–45	400	0.012

*^*x*^Growth rates were estimated by dividing maximum length by root age. It was assumed that maximum length of all roots was achieved by the oldest of their class and that branched nodal roots formed from 2 DAE, fast nodal roots from 7 DAE, and L-type roots from 4 DAE.*

Lateral roots varied in length and, presumably, in growth rates, with L-type and S-type lateral roots having developed on the primary root being 10% longer compared to roots from a branched nodal root. Similarly, root hairs were longer on the primary root (0.02 cm) compared to crown and L-type laterals (0.015 cm). Root hairs on S-type laterals were shorter (0.012 cm) and the maximum density was lower (400 hairs cm^–1^) compared to L-type lateral (700 hairs cm^–1^) and nodal roots (1,440 hairs cm^–1^). Based on observations by Nestler and Wissuwa (2016), it was assumed that the proportion of roots being alive is decreasing over time and this was modeled as a linear decreasing root hair density over time (e.g., from 600 hairs cm^–1^ on a 3-day old root to 330 hairs cm^–1^ on a 40-day old root).

**FIGURE 2:**
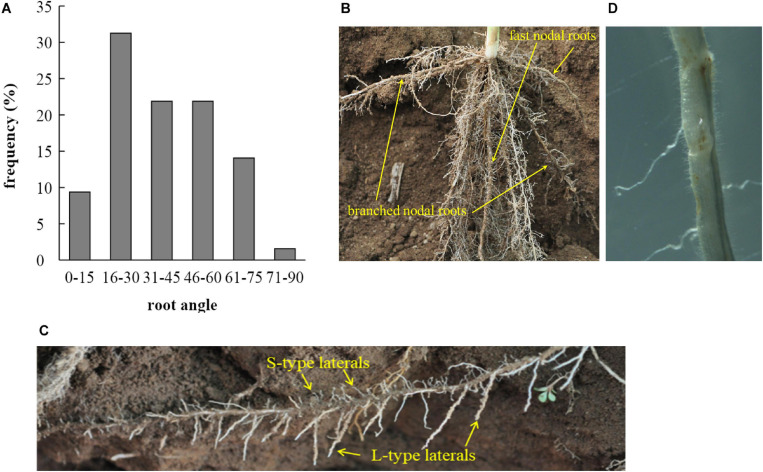
**(A)** Distribution of root growth angles (RGA, in degree from the soil surface) of nodal roots excavated 28 DAE from the field experiment and **(B)** partially excavated root system used to determine RGAs. The fast nodal roots are indicated in comparison to their branched counterparts. **(C)** A branched nodal root growing at a shallow angle developing a large number of L-type and S-type laterals. **(D)** Micrograph of an L-type lateral root section with S-type lateral roots and root hairs.

Measurements of root length and depths, taken during excavations in the field at 28 DAE, provided data for root growth angles (RGA) of crown roots. These varied between 8 and 90 degrees (from the soil surface), with most roots having RGAs between 16 and 30° (Figure 2A; *n* = 64). Overall, 62% of nodal roots had an RGA ≤ 45°. The distribution of RGAs appears to be steeper in Figure 2B than measured data would suggest (Figure 2A) but this is caused by roots growing toward the observer having been fully excavated and thereby losing their original root angle.

L-type and S-type lateral roots were assumed to develop perpendicular to their root of origin and their growth direction would therefore depend on where on the parent root they were initiated. The distribution of root development along the branched nodal root partially excavated from the field and shown in Figure 2C would suggest that the L-type lateral roots preferentially develop or extend on the downward side of nodal roots whereas S-type laterals appear as frequently on the upper half. This was simulated in OpenSimRoot by setting a boolean command named “cannotgrowup” which ‘corrects’ the growth direction of upward growing roots to horizontal. The L and S type roots have no gravitropism modifier, whereas the primary root has a strong (−0.115 to −0.015 cm/cm/day) gravitropism, and crown roots a weak (−0.0001 cm/cm/day) gravitropism.

### Comparison of Simulated and Observed Root Growth of DJ123

**FIGURE 3:**
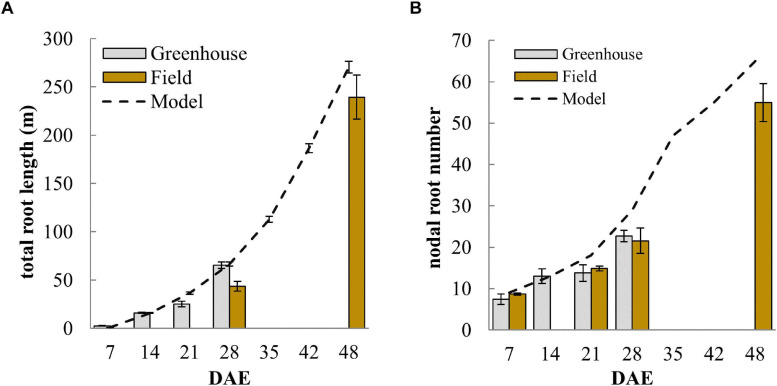
Development of **(A)** nodal root number and **(B)** total root length over time comparing measured values from greenhouse and field experiments with simulated data from the model. Error bars represent standard deviations (*n* = 4).

The total root length (TRL) simulated by the model is the result of the various model inputs and shall thus be used to verify that simulated root system development is reflecting the observed root growth. The model slightly over-estimated TRL at 21 DAE and again at 48 DAE but was in-between the observed TRL from greenhouse and field experiments at 28 DAE (Figure 3A). The biggest deviation form simulated total root length was seen in the field sample at 28 DAS and this may have been due to slower early root development in the field during the earlier part of the season when lower night-time temperatures may have limited growth. A main difficulty in modeling root development during the first 3 to 4 weeks stemmed from the observation that nodal root emergence was non-linear. Nodal root emergence was rapid during the first 7 to 10 days and that this was followed by a slower increase in nodal root number during the next 7 to 10 days (Figure 3B). Thus, root development during the first 28 days neither followed a purely linear nor exponential growth trend. The model matched this initial slowdown (Figure 3B) by slowing the rate of nodal root formation from day 10. The slowing down might be associated with the depletion of seed reserves that supported rapid root development initially.

### Simulation of Root Hairs and Effects on Total Root Length and P Uptake

**FIGURE 4:**
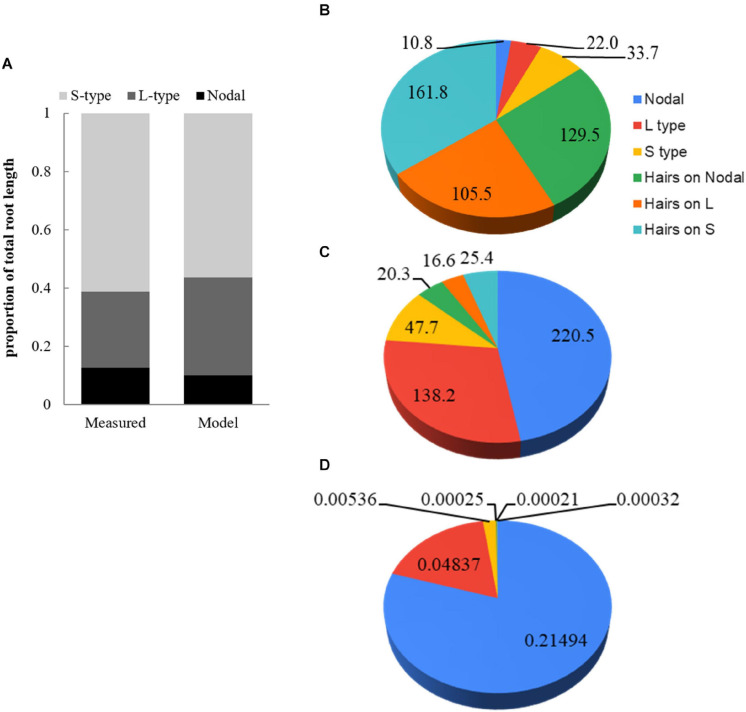
Comparison of root class distributions between **(A)** measured and model data and **(B)** modeled contribution (%) by root classes including root hairs developing on respective classes for total root length, **(C)** surface area, and **(D)** volume.

At 28 DAE the WinRhizo analysis of root scans indicated that about 61% of the total root length was contributed by S-type lateral roots, followed by 26% for L-type lateral and only 13% for nodal roots including the primary root (Figure 4A). Modeled proportions were in general agreement, but L-type laterals had increased to 33.6% with ensuing minor reductions of other classes. Furthermore, simulations allowed us to compare the total length of root hairs to the length of the root system and individual root classes. The total length of all root hairs is 6.9-time the length of the root system. Root hairs on S-type lateral roots are short (0.12 mm) and present in lower density compared to L-type and nodal roots, nevertheless we estimated that 29.7% of the total root hair length is due to hairs on S-types (Figure 4B). The proportion of total root hair length on L-type laterals is 38.7%. Hairs on all root classes combined have an estimated length of 482.3 m compared to 52.9 m for the actual roots. Due to the small diameter of root hairs (5 μm) their contribution to total root surface area is only 13.3% (Figure 4C). They only constitute 1.8% of total root volume (Figure 4D).

### Simulation of P Uptake

**FIGURE 5:**
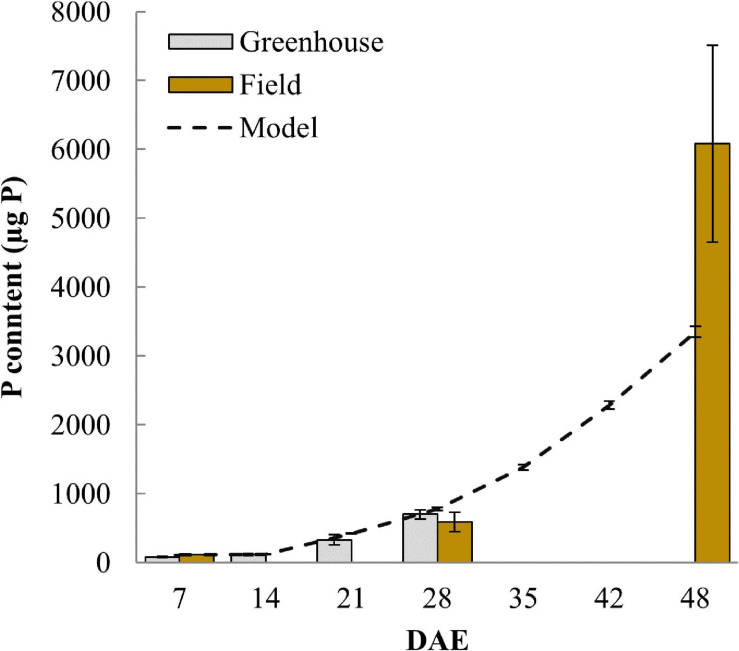
Plant P content over time comparing measured values from greenhouse and field experiments with simulated data from the model. Error bars represent standard deviations (*n* = 4).

P uptake was initially modeled using the measured soil solution P concentration of 83 nM and that resulted in a plant P content of only 150.6 μg at day 28 (data not shown), compared to a measured P content of 774.3 μg. Such underestimation of P uptake under highly P deficient conditions is commonly observed in P uptake models, indicating that more P is typically available to the plant than suggested by soil P extractions (Kirk et al., 1999). To account for the higher measured P uptake, we assumed that the roots, possibly through pH changes or some other rhizosphere modification, can solubilize P thereby increasing the P concentration in soil solution. Adopting a soil solution P concentration of 540 nM increased simulated P uptake to within standard deviations of measured P content at 21 and 28 DAE (Figure 5). All further simulations are using this higher soil P concentration in order to generate simulated P uptake that is comparable to observed uptake. However, even with this higher P concentration the model continued to underestimate P content in the field at 48 DAE (Figure 5) and this is because the P taken up per unit root length increased in the field to 26.2 μg P m^–1^ from 12.1 μg P m^–1^ at 28 DAE (Supplementary Figure 1). Respective values simulated by the advection-diffusion model implemented in OpenSimRoot were almost constant, only changing from 11.6 to 12.4 μg P m^–1^ between 28 and 48 DAE.

### Sensitivity Analysis and Cost-Benefit Simulations

The sensitivity analysis estimated to what extent P uptake was affected by changes in model input parameters. Variations in soil P concentrations across the entire 16-fold range from 25% to 400% showed a near-linear and proportional effect on P uptake (Table 2). Changes in soil buffer (b) and the corresponding changes in the diffusion coefficient (De) were less influential and seemed to be more sensitive to large increases than to proportionally large decreases. Effects of altering lateral root length and density were also assessed through changing the “inter-branching distance” (IBD). Greater IBD leads to fewer lateral roots and, consequently, less uptake. Changes in lateral root length and density were more influential in L-type than S-type lateral roots and increasing L-type length was most effective in increasing P uptake (Table 2). The model was less sensitive to proportional decreases than to increases in lateral root length and density. Similar effects were detected for root hair length, but simulated P uptake was rather insensitive to changes in root hair density.

**TABLE 2:** Sensitivity analysis for two soil parameters (P concentration in soil solution and soil buffer power) and six root parameters (length and density for L-type roots, S-type roots, and root hairs).

P concentration in soil solution			Soil buffer power		
Factor level	P content (μg)	Change (%)	Factor level	P content (μg)	Change (%)
0.25	416	30	0.25	866	62
0.5	732	53	0.5	1,071	77
1	1,391	100	1	1,391	100
1.5	2,029	146	1.5	1,622	117
2	2,659	191	2	1,827	131
4	5,087	366	4	2,738	197

**L-type length**			**S-type length**		
**Factor level**	**P content (μg)**	**Change (%)**	**Factor level**	**P content (μg)**	**Change (%)**

0.25	947	68	0.25	1,160	83
0.5	1,080	78	0.5	1,221	88
1	1,391	100	1	1,391	100
1.5	1,696	122	1.5	1,520	109
2	1,995	143	2	1,678	121
4	3,301	237	4	2,273	163

**L-type branching distance**			**S-type branching distance**		
**Factor level**	**P content (μg)**	**Change (%)**	**Factor level**	**P content (μg)**	**Change (%)**

4	974	70	4	1,155	83
2	1,100	79	2	1,230	88
1.5	1,196	86	1.5	1,282	92
1	1,391	100	1	1,391	100
0.5	1,925	138	0.5	1,669	120
0.25	3,042	219	0.25	2,232	161

**Root hair length**			**Root hair density**		
**Factor level**	**P content (μg)**	**Change (%)**	**Factor level**	**P content (μg)**	**Change (%)**

0.25	1,190	86	0.25	1,222	88
0.5	1,215	87	0.5	1,278	92
1	1,391	100	1	1,391	100
1.5	1,624	117	1.5	1,481	107
2	1,899	137	2	1,545	111
4	3,168	228	4	1,804	130

*Parameters were varied by a factor 0.25×, 0.5×, 1.5×, 2.0×, and 4×, with factor level 1 representing the base model. The sensitivity analysis estimated effects of parameter variations on P uptake for a 28 day period (25 DAE). Data shown are means of 5 repeat simulations.*

When plant growth is directly limited by P availability, any new tissue produced incurs a cost in term of P allocated to the respective tissue and this can be compared to the benefit this root provides in terms of P uptake. P uptake over a 28-day growth period was far higher in nodal roots compared to L-type and S-type lateral roots, and this was associated with the larger surface area nodal roots produced, despite having much lower total length (Table 3). Our simulations further showed that more than 80% of the total root system weight was due to nodal roots, which also meant that they contributed a similarly high share to the cost of a root system in terms of P invested. Root hairs on the other hand were negligible in terms of cost but increased P uptake by 19%, adding 22% of extra surface area and sixfold extra length (Table 3).

**TABLE 3:** Model simulation results by root class (and root hairs) showing root dimensions, costs in terms of P needed to produce roots, and benefits in terms of P taken up.

	P uptake	Total root				P uptake efficiency			Benefit to cost	Cost recovery		
		Length	Surface	Weight	Cost	Length	Surface	Weight		Case 1	Case 2	Case 3

	μg	m	cm^2^	mg	μg P	μg m^–1^	μg cm^–2^	μg mg^–1^	ratio	days	days	days
Hairs	110.0	395.9	62.2	0.78	0.6	0.3	1.77	141.4	176.8			
S-type	107.4	33.7	47.7	4.6	3.7	3.2	2.25	23.1	28.9	0.4	1.0	1.0
L-type	174.5	21.9	138.2	50.5	40.4	7.9	1.26	3.5	4.3	2.7	4.8	5.1
Nodal	297.4	10.8	220.5	399.9	319.9	27.6	1.35	0.7	0.9	12.5	24.8	Never
Total	689.2	462.4	468.6	455.9	364.7	1.5	1.47	1.5	1.9			

*Costs were estimated based on a P concentration of 0.8 mg g^–1^ in all root classes including hairs. Data shown is for 31 days after germination (equivalent to 28 DAE in field and greenhouse experiments). The cost recovery in days was estimated for three cases. Case 1 is forward looking, estimating the time needed to recover the cost of a respective root class based on static costs and P uptake rates at 28 DAE. Case 2 and 3 are based on the cumulative costs and P uptake, starting at the time point a respective root class had emerged and simulating the days needed until cumulative P uptake has surpassed cumulative costs. For case 2 root hairs are included on the respective root classes whereas case 3 simulated P uptake without root hairs.*

When P uptake efficiency was estimated as P taken up per root length, main root axes were more efficient due to their larger diameter and surface area, however, per root weight finer roots and especially root hairs were far more efficient. When P uptake efficiency was estimated based on root surface area, root classes were rather similar with a slight efficiency advantage for S-type lateral roots. As a result of their very low cost, roots hairs had the best benefit to cost ratio, and we estimated that over the 31 day growth period, their cumulative P uptake exceeded their cost (P invested in producing them) by a factor 176.8 (Table 3). Based on the cost of a root class and the daily P uptake of that root class (static – both at 28 DAE) the number of days needed to recover that cost was estimated (Case 1, Table 3), suggesting that S-type lateral roots recover their own cost on the day of their emergence, whereas a nodal root would require more than 12 days. Using a different cost-recovery scenario that estimates the break-even time point of a hypothetical developing root system made up entirely of one single root class puts nodal roots even more at a disadvantage relative to lateral roots (Case 2, Table 3). If one would simulate a root system without root hairs (Case 3) a continuously growing nodal root system would never reach the point where P uptake exceeds the P cost of producing the root system.

To test to what extent these large differences in benefit to cost ratio are affected by the model root parameterization, additional simulations were conducted with hypothetical root systems having reduced or increased S-type and L-type lateral root length or reduced nodal root diameter. Halving or doubling lateral root length had only minor effects on benefit-cost ratios (Supplementary Table 4). More influential was a decrease in diameter of fast nodal roots (from 0.65 to 0.50 cm), offering over-proportionally high cost savings of 25% while lowering P uptake by only 8.2% (Supplementary Table 4). While this meant nodal roots changed from a net-sink to a source of P, benefit-cost ratios of other root types remained several-fold higher.

## Discussion

The potential utility of root architectural models in conceptualizing factors determining resource acquisition has long been recognized. Here our objective was to develop a 3D root architectural model for upland rice, using root developmental parameters obtained by stepwise excavation of partial root systems of field grown plants or of entire seedling root systems from experiments conducted in containers large enough to not restrict root development of young plants. A further step to simulate an upland rice root system as realistically as possible was the inclusion of root hairs as individual cylinders of given length and diameter, as opposed to simply expanding the overall radius of the parent root by the root hair length. Parameters of root hair length and density were adopted from Nestler et al. (2016), Nestler and Wissuwa (2016) who showed root hairs to exist even on S-type lateral roots. They furthermore showed root hair length to differ between root classes and to be substantially shorter in soil than in nutrient solutions. These findings have been included in the specification of root hair parameters for the first time in this model.

### Modeling Root Development and 3D Root Architecture

Our model was able to simulate the root development of our model genotype DJ123 closely in terms of total crown roots or total root system length produced (Figure 3). The image presented in Figure 1 furthermore showed that crown roots developed at different angles from shallow to near-vertical. Our model estimated 87% of the roots to be present in the top 25 cm (data not shown) and this compares to 79% of root biomass in the top 25 cm detected in root excavations by Mori et al. (2016). Roots in that study were from 105-day old plants, which would have developed a higher proportion of deep roots compared to our simulated plant at 28 DAE.

In excavating roots we could visibly distinguish 2 classes of crown roots based on their thickness and the degree of branching. We have termed these branched and long nodal roots and to our knowledge such a distinction has not been made before. Nodal roots developing on young seedlings were of the branched class and as the plant matured, an increasing proportion of crown roots were of the faster and longer class. We hypothesized that such a change in predominant class is related to inter-root competition for resources as root zones may increasingly overlap closer to the point of origin. Developing fast extending roots with little branching in the first 10 cm would be a way to limit placing resources in that overcrowded soil volume, however, model simulations did not support this hypothesis as overlapping depletion zones were found to be generally low and hardly affected by the presence or absence of the fast nodal root type (Supplementary Figure 2). Thus, the main advantage of rapidly extending roots into yet to be explored soil would presumably be to maximize exploration for water and nutrients and that fast nodal roots did not develop in young seedlings may simply be a reflection of their larger diameter which incurs a higher cost in terms of P and biomass. In combination with less branching it would take longer to recover that cost compared to branched crown roots and this could be more of a limiting factor in younger plants.

### Modeling Total P Uptake Over Time

Using the extremely low measured soil P concentrations (0.083 nmol mL^–1^) in modeling P uptake severely underestimated the actual measured P uptake in DJ123. Such underestimation is common in Barber-Cushman-based models if soil P availability is very low (Kirk et al., 1999), indicating that some processes that increase P availability, such as pH effects, root exudation, or soil microbial effects, are not properly accounted for in the model. We acknowledge that such processes should ideally be addressed in future models, but that is beyond the scope of the present study primarily focusing on contributions of different roots class to overall P uptake. We solved the underestimation of P uptake by increasing soil P concentrations to a level (540 nM) where P uptake matched observed uptake. While this may appear arbitrary, it allows us to model within the range of observed P uptake without making further assumptions about specific but untested P solubilization mechanisms.

The soil P concentrations used enabled us to closely match observed P uptake of field and greenhouse experiments up to 28 DAE, however, at 48 DAE field P uptake remained 81% higher compared to our modeled uptake. We hypothesize that this may be due to additional P provided through the arbuscular mycorrhiza (AM) pathway. Using the same soil Wissuwa et al. (2020) showed that roots of DJ123 and other rice genotypes started to be colonized by AM from 21 DAE onward and that the symbiosis was active by 28 DAE without having contributed much additional P until then. It is likely that P provided through the AM pathway would increase with time in the field and may therefore add significantly to total plant P content at 48 DAE. If our hypothesis is true, at 48 days, as much as 40% of P uptake might have come from VAM. We note, however, that other mechanisms may contribute to additional P uptake as well, including slow release of P, mineralization of P, or solubilization through slowly changing rhizosphere pH.

### Modeling P Uptake by Root Class and Cost-Benefit Analysis

In simulations of P uptake with and without root hairs present, we could estimate the contribution of root hairs and other root classes to P uptake. Most P (43%) is taken up by the nodal roots with L-type laterals also contributing substantially (25%), whereas S-type lateral roots and root hairs would only contribute around 15% each (Table 3). Their advantage becomes obvious, however, when P uptake is examined in relation to the cost of producing roots of a certain class. Due to their minute biomass, we estimated that root hairs have taken up 176-times more P than was necessary to construct them (at 28 DAE). P uptake to investment ratios were also high for S-type lateral roots and simulating different root system architectures showed that the cost-benefit advantage of root hairs and S-type lateral roots over other root types was very consistent (Supplementary Table 4). The high cost of producing nodal roots meant that their direct P uptake did not fully recover their cost. This could be reversed by reducing the crown root diameter, which incurred savings in terms of P cost that were greater than losses in P taken up. However, to what extent reduced crown root diameter would compromise water uptake needs to be considered. A slightly different way of evaluating cost and benefits would be to consider the cost recovery time. Despite some differences between cases 1 to 3, the common picture emerging from the cost recovery analysis was that low-cost S-type lateral roots and root hairs break even within 1 day, which would make them the root structure of choice for a root system adapted to P deficiency. The much later break-even point for crown root would furthermore explain why crown root number is very strongly reduced under P deficiency (Wissuwa et al., 2020).

### Optimizing the Root System

A question of practical relevance in plant breeding is related to the choice of traits to be targeted in selection in order to maximize varietal performance. In the context of the present study one may explore different root classes and their properties as likely targets for selection. The sensitivity analysis identified L-type lateral root length and root hair length as the two most influential parameters. That the length of L-type lateral roots was more influential compared to S-type length was likely due to the fact that longer L-types automatically produce more S-type laterals, assuming a constant branching frequency of S-types on L-types. This would be consistent with the conclusion by De Bauw et al. (2020) that total P uptake was dependent on the number of total root tips, which in turn depends on the rate of secondary branching on L-type laterals. Thus, producing higher-order roots would seem advantageous due to the knock-on effect on increasing subsequent roots classes and root hairs. However, the cost-benefit analysis indicated higher-order roots are less effective per P invested, which would favor the formation of root hairs and S-type lateral roots as traits for better adaptation to more severe P deficiency. This would also hold true in relation to cost per root tip as S-type roots are by far the most numerous root type. A point to be addressed in future studies is to what extent the process of continuous root tip growth into yet unexplored soil is crucial (De Bauw et al., 2020), as S-type roots will reach their maximum length of around 1 cm faster compared to longer L-type laterals.

The sensitivity analysis showed that increasing the length of root hairs was far more beneficial for P uptake compared to increasing root hair density. Employing a very different modeling approach Zygalakis et al. (2011) reached the same conclusion. Should root hair length therefore be a primary target trait in rice improvement? This depends to what extent genotypic differences in root hair extension will be realized in soil. Nestler et al. (2016) reported average root hair length of 430 μm on nodal roots grown in low-P nutrient solution, which decreased to below 200 μm on soil-grown roots. *In situ* synchrotron images of a soil-grown root with 243 visible root hairs furthermore clarified that a maximum length of 395 μm could be reached by hairs developing into larger soil pores, but that the average length of root hairs was a mere 122 μm due to the physical barrier imposed by soil particles (Nestler et al., 2016) and the inability of hairs to bend and grow around obstacles. Thus, a crucial question to be resolved is to what extent potential genotypic differences in root hair length would be realized in the field and whether genotypic differences in the ability to extend root hairs against soil resistance exist.

Unlike root hairs, S-type lateral roots can modify their growth direction (see Figure 2D) and can therefore elongate around soil particles, and selection for increased S-type length would be the second-most efficient way to increase P uptake per cost. To our knowledge, efforts to systematically evaluate genotypic differences in S-type lateral root length have not been made. One interesting aspect of our cost-benefit analysis regarding S-type lateral roots is that they become a net-source of P after only 1 day of existence, much earlier compared to larger root classes. What remains unknown is for how long these S-type roots remain active and contribute to uptake of P and other resources. Their relative advantage would diminish if their active life span was very limited and further investigations clarifying this point are needed. As for S-type length, any genotypic difference in longevity would potentially be worth exploiting in rice breeding.

## Conclusion

We constructed a detailed model of the root architecture and morphology of upland rice, using DJ123 as a representative of upland rice genotypes with adaptations to low P soils. Modeling its root system with S-type lateral roots more than doubled root system length and adding root hairs further increased total length several-fold. Despite the presence of such extensive length in fine structures, P uptake attributable to these structures was only around 30% because the corresponding surface area was small. The significance of the fine root structures characterizing an upland rice root system appear to be related to their low cost. Under severe P deficiency root growth is reduced, presumably by the growth-limiting factor P. P invested in extending a root system therefore represents a crucial cost and the rapid recovery of that cost by S-type lateral roots and root hairs may enable rice to continuously expand its root system, thereby maintaining P uptake.

## Data Availability Statement

The model program code including input data used has been made publicly available at https://github.com/GDanielGzz/OpenSimRootDJ123.

## Author Contributions

DG did the measurements and parameterizations for the model, developed the model itself, and wrote the manuscript. JP created the base program used for creating the model, provided technical support and advice, and edited the manuscript. MW advised and overviewed the development of the model, and provided assistance in writing and editing the manuscript. All authors contributed to the article and approved the submitted version.

## Conflict of Interest

The authors declare that the research was conducted in the absence of any commercial or financial relationships that could be construed as a potential conflict of interest.

## References

[B1] Brown L. K. George T. S. Thompson J. A. Wright G. Lyon J. Dupuy L. (2012). What are the implications of variation in root hair length on tolerance to phosphorus deficiency in combination with water stress in barley (*Hordeum vulgare*)? *Ann. Bot.* 110 319–328. 10.1093/aob/mcs085 22539540 PMC3394649

[B2] De Bauw P. Mai T. H. Schnepf A. Merckx R. Smolders E. Vanderborght J. (2020). A functional-structural model of upland rice root systems reveals the importance of laterals and growing root tips for phosphate uptake from wet and dry soils. *Ann. Bot.* 126 789–806. 10.1093/aob/mcaa120 32597468 PMC7489101

[B3] Diagne A. Amovin-Assagba E. Koichi F. Wopereis M. C. S. (2013). “Estimation of cultivated area, number of farming households and yield for major rice-growing environments in Africa,” in *Realizing Africa’s Rice Promise*, eds Wopereis M. C. S. Johnson D. E. Ahmadi N. Tollens E. Jalloh A. (Boston, MA: CABI), 35–45.

[B4] Drame N. K. Manneh B. (2013). “Rice genetic improvement for abiotic stress tolerance in Africa,” in *Realizing Africa’s Rice Promise*, eds Wopereis M. C. S. Johnson D. E. Ahmadi N. Tollens E. Jalloh A. (Boston, MA: CABI), 144–160.

[B5] Fageria N. K. Moreira A. dos Santos A. B. (2013). Phosphorus uptake and use efficiency in field crops. *J. Plant Nutr.* 36 2013–2022. 10.1080/01904167.2013.816728

[B6] Freschet G. T. Roumet C. Comas L. H. Weemstra M. Bengough A. G. Rewald B. (2020). Root traits as drivers of plant and ecosystem functioning: current understanding, pitfalls and future research needs. *New Phytol.* 10.1111/nph.17072 33159479

[B7] Gahoonia T. S. Nielsen N. E. (1998). Direct evidence on participation of root hairs in phosphorus (32P) uptake from soil. *Plant Soil* 198 147–152. 10.1023/A:1004346412006

[B8] IRRI World Rice Statistics (2020). *Rice Yields in Asia.* Available online at: http://ricestat.irri.org:8080/wrsv3/entrypoint.htm (accessed July 26, 2019).

[B9] Kirk G. L. D. Santos E. E. Findenegg G. R. (1999). Phosphate solubilization by organic anion excretion from rice (*Oryza sativa* L.) growing in aerobic soil. *Plant Soil* 211 11–18.

[B10] Mori A. Fukuda T. Vejchasarn P. Nestler J. Pariasca-Tanaka J. Wissuwa M. (2016). The role of root size versus root efficiency in phosphorus acquisition in rice. *J. Exp. Bot.* 67 1179–1189. 10.1093/jxb/erv557 26842979

[B11] Morita S. Abe J. (1994). “Modeling root system morphology in rice,” in *Biology of Adventitious Root Formation*, eds Davis T. D. Haissig B. E. (Boston, MA: SSBM). 10.1007/978-1-4757-9492-2_15

[B12] Murphy J. Riley J. P. (1962). A modified single solution method for the determination of phosphate in natural waters. *Anal. Chim. Acta* 27 31–36. 10.1016/S0003-2670(00)88444-5

[B13] Nestler J. Keyes S. D. Wissuwa M. (2016). Root hair formation in rice (*Oryza sativa* L.) differs between root types and is altered in artificial growth conditions. *J. Exp. Bot.* 67 3699–3708. 10.1093/jxb/erw115 26976815

[B14] Nestler J. Wissuwa M. (2016). Superior root hair formation confers root efficiency in some, but not all, rice genotypes upon P deficiency. *Front. Plant Sci.* 7:1935. 10.3389/fpls.2016.01935 28066487 PMC5174101

[B15] Nigatu G. Hansen J. Childs N. Seeley R. (2017). Sub-Saharan Africa is projected to be the leader in global rice imports. *Amber Waves* 9:17. 10.22004/AG.ECON.266026

[B16] Postma J. A. Kuppe C. Owen M. R. Mellor N. Griffiths M. Bennett M. J. (2017). OpenSimRoot: widening the scope and application of root architectural models. *New Phytol.* 215 1274–1286. 10.1111/nph.14641 28653341 PMC5575537

[B17] Rebouillat J. Dievart A. Verdeil J. L. Escoute J. Giese G. Breitler j-c, et al. (2009). Molecular genetics of rice root development. *Rice* 2 15–34. 10.1007/s12284-008-9016-5

[B18] Rose T. J. Impa S. M. Rose M. T. Pariasca-Tanaka J. Mori A. Heuer S. (2013). Enhancing phosphorus and zinc acquisition efficiency in rice: a critical review of root traits and their potential utility in rice breeding. *Ann. Bot.* 112 331–345. 10.1093/aob/mcs217 23071218 PMC3698374

[B19] Saito K. Vandamme E. Johnson J. M. Tanaka A. Senthilkumar K. Dieng I. (2019). Yield-limiting macronutrients for rice in sub-Saharan Africa. *Geoderma* 338 546–554. 10.1016/j.geoderma.2018.11.036

[B20] Silberbush M. Barber S. (1984). Phosphorus and potassium uptake of field-grown soybean cultivars predicted by a simulation model. *Soil Sci. Soc. Am. J.* 48 592–596.

[B21] Silberbush M. Barber S. (1983). Sensitivity of simulated phosphorus uptake to parameters used by a mechanistic-mathematical model. *Plant Soil* 74 93–100.

[B22] Tsujimoto Y. Rakotoson T. Tanaka A. Saito K. (2019). Challenges and opportunities for improving N use efficiency for rice production in sub-Saharan Africa. *Plant Prod. Sci.* 22 413–427. 10.1080/1343943X.2019.1617638

[B23] Vandamme E. Rose T. Saito K. Jeong K. Wissuwa M. (2015). Integration of P acquisition efficiency, P utilization efficiency and low grain P concentrations into P-efficient rice genotypes for specific target environments. *Nutr. Cycl. Agroecosyst.* 104 413–427. 10.1007/s10705-015-9716-3

[B24] Wissuwa M. Ae N. (2001). Genotypic variation for tolerance to phosphorus deficiency in rice and the potential for its exploitation in rice improvement. *Plant Breed.* 120 43–48. 10.1046/j.1439-0523.2001.00561.x

[B25] Wissuwa M. Gonzalez D. Watts-Williams S. J. (2020). The contribution of plant traits and soil microbes to phosphorus uptake from low-phosphorus soil in upland rice varieties. *Plant Soil* 448 523–537. 10.1007/s11104-020-04453-z

[B26] Wissuwa M. Kondo K. Fukuda T. Mori A. Rose M. T. Pariasca-Tanaka J. (2015). Unmasking novel loci for internal phosphorus utilization efficiency in rice germplasm through Genome-Wide Association Analysis. *PLoS One* 10:e0124215. 10.1371/journal.pone.0124215 25923470 PMC4414551

[B27] Zygalakis K. C. Kirk G. J. D. Jones D. L. Wissuwa M. Roose T. (2011). A dual porosity model for nutrient uptake by root hairs. *New Phytol.* 192 676–688. 10.1111/j.1469-8137.2011.03840.x 21827499

