# Supplementary material for: Cost-Benefit Analysis of the Upland-Rice Root Architecture in Relation to Phosphate: 3D Simulations Highlight the Importance of S-Type Lateral Roots for Reducing the Pay-Off Time
Source: Front Plant Sci. 2021 Mar 12;12:641835. doi: 10.3389/fpls.2021.641835 (PMC7996052; doi:10.3389/fpls.2021.641835)
Supplement: Supplementary file 1 [file Table_1.docx]

| supplementaryTable S1 Soil parameters used in the model based on analyses by Rakotoson (unpublished) | | |
| --- | --- | --- |
| P concentration in soil solution | 83 | nM |
| Soil Diffusion (De) | 0.00000922752 | cm^2^ day^-1^ |
| Buffer Power (b) | 6000 | Dm^3^ |
| Adsorption Coefficient () | 1333.3 | µmol cm^-1^ |
| Saturated Diffusion Coefficient () | 0.00495 | cm^2^ day^-1^ |
| H2PO4-Diffusion coefficient in water (Di) | 0.7344 | cm^2^ day^-1^ |
| Soil volumetric Water Content (θ) | 0.3 | Dm^3^ |
| Diffusion impedance factor (tortuosity factor) (f) | 0.2 | N/A |
