# Supplementary material for: Cost-Benefit Analysis of the Upland-Rice Root Architecture in Relation to Phosphate: 3D Simulations Highlight the Importance of S-Type Lateral Roots for Reducing the Pay-Off Time
Source: Front Plant Sci. 2021 Mar 12;12:641835. doi: 10.3389/fpls.2021.641835 (PMC7996052; doi:10.3389/fpls.2021.641835)
Supplement: Supplementary file 5 [file Presentation_1.pptx]

## Slide 1
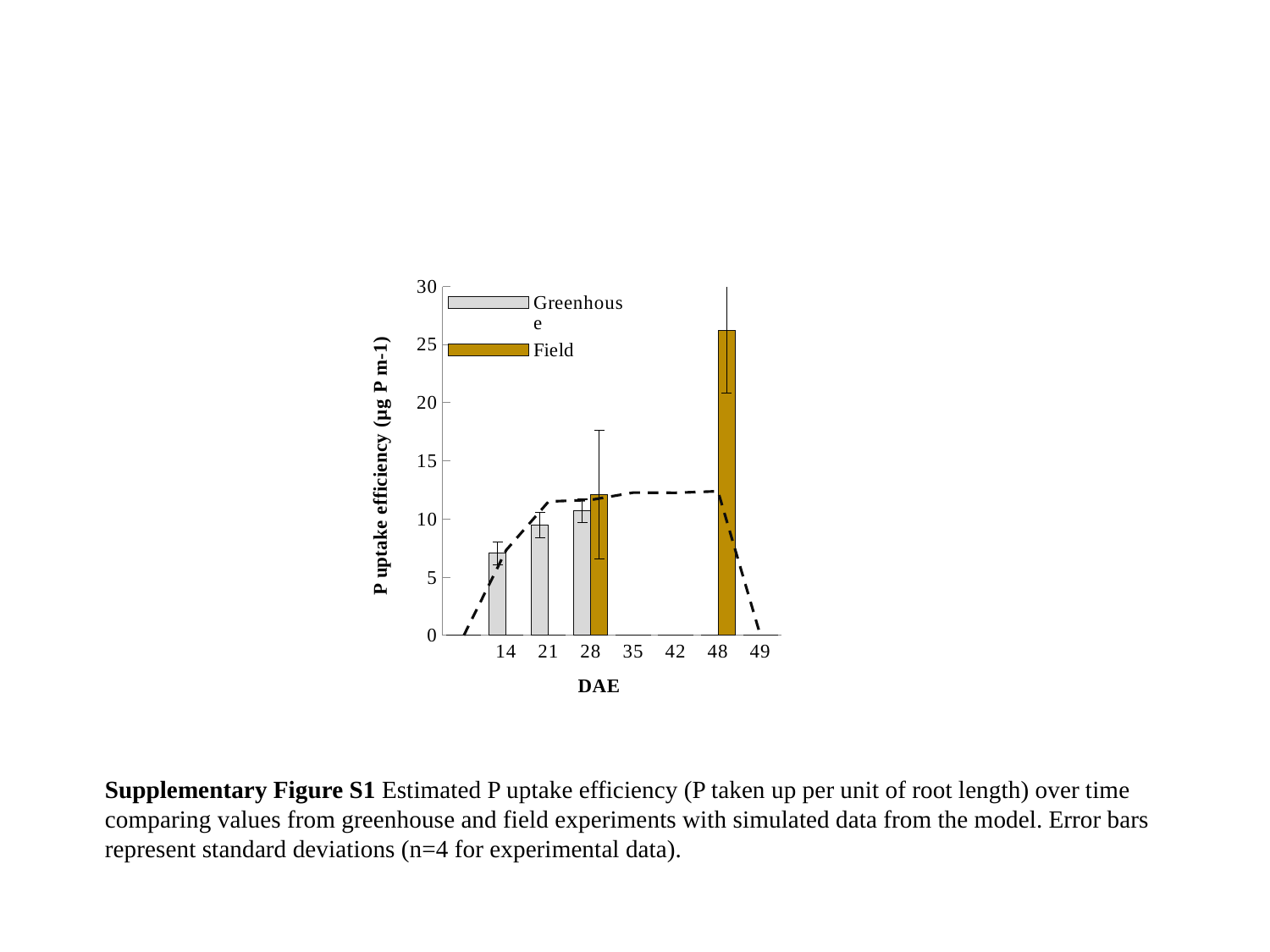

[unsupported chart]
Supplementary Figure S1 Estimated P uptake efficiency (P taken up per unit of root length) over time comparing values from greenhouse and field experiments with simulated data from the model. Error bars represent standard deviations (n=4 for experimental data).
