# Supplementary material for: Cost-Benefit Analysis of the Upland-Rice Root Architecture in Relation to Phosphate: 3D Simulations Highlight the Importance of S-Type Lateral Roots for Reducing the Pay-Off Time
Source: Front Plant Sci. 2021 Mar 12;12:641835. doi: 10.3389/fpls.2021.641835 (PMC7996052; doi:10.3389/fpls.2021.641835)
Supplement: Supplementary file 6 [file Presentation_2.pptx]

## Slide 1
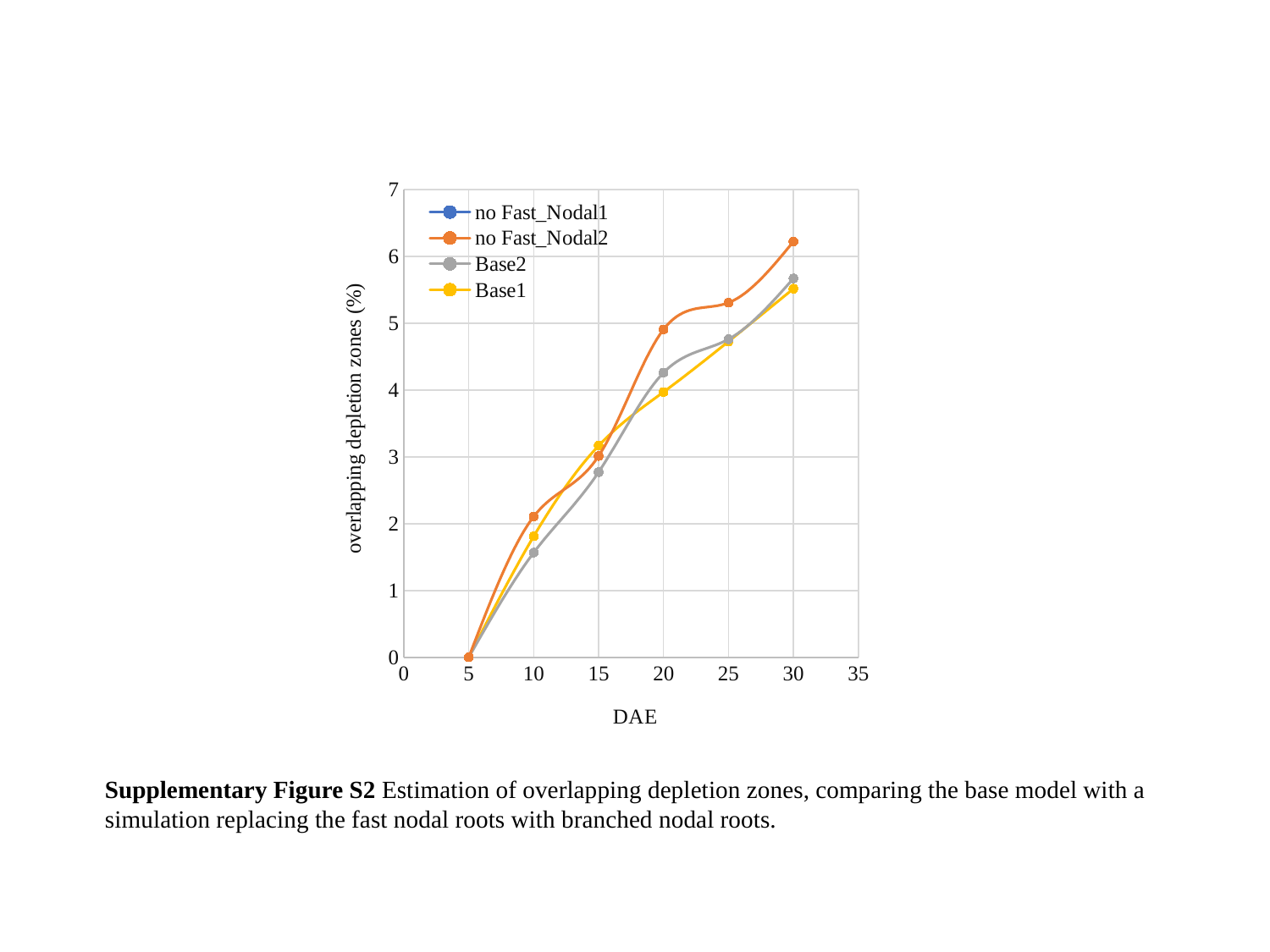

### Chart
| Category | | | | |
|---|---|---|---|---|Supplementary Figure S2 Estimation of overlapping depletion zones, comparing the base model with a simulation replacing the fast nodal roots with branched nodal roots.
